# SARS‐CoV‐2 first contact: Spike–ACE2 interactions in COVID‐19

**DOI:** 10.1111/cbdd.13898

**Published:** 2021-06-21

**Authors:** Salvatore Nesci

**Affiliations:** ^1^ Department of Veterinary Medical Sciences University of Bologna Bologna Italy

## Abstract

The full‐length human ACE2 in complex with B^0^AT1 is anchored to two *S* in open pre‐fusion state which allows establishing pre‐invasion interactions with the ACE2 N‐terminal domain.

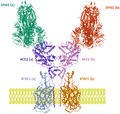

Nowadays, a severe acute respiratory syndrome and pneumonia named COVID‐19 has emerged as a serious pandemic (Wu et al., 2020; Zhou et al., 2020) caused by a new coronavirus named severe acute respiratory syndrome coronavirus 2 (SARS‐CoV‐2; Wu et al., 2020; Zhou et al., 2020). Recently, the Cryo‐EM structure of three receptor‐binding domains (RBDs) of the SARS‐CoV‐2 spike (*S*) in receptor‐accessible conformation is obtained (Walls et al., 2020; Wrapp et al., 2020). Accordingly, the docking of RBD *S* onto the angiotensin‐converting enzyme 2, known as ACE2, of host cells allows facing the therapeutic treatment of SARS‐CoV‐2 infection (Lan et al., 2020; Yan et al., 2020). Indeed, the virus anchors the host cells by the interaction of the *S* protein with the human ACE2 receptor, triggering the pre/postfusion conformational change responsible for the virus entry into the host cell (Mercurio et al., 2021). Each protomer of *S* glycoprotein is composed of S_1_ and S_2_ subunits. The S_1_ subunit includes N‐terminal domain and the RBD bearing the receptor recognition/attachment site, and the S_2_ subunit includes the C‐terminal domain of glycoprotein responsible for viral and cellular membrane fusion. The *S* glycoprotein prefusion state undergoes conformational changes after a cleavage event, led by host proteases, occurring at the furin‐binding region. The furin cleavage site is located at the boundary between the S_1_ and S_2_ subunits. After protease cut, the S_2_ refolding induces *S* postfusion conformation. In the prefusion conformation, the three protomers of *S* undergo two different states with high flexibility (Mercurio et al., 2021). Indeed, the RBDs of the S_1_ subunit can move from ‘RBD down’ to ‘RBD up’ conformation. The former corresponds to the receptor‐inaccessible binding site, and the latter is the receptor‐accessible binding site. However, the predominant state of *S* glycoprotein in the prefusion conformation has two protomers in ‘RBD down’ and one in ‘RBD up’ conformation (Pierri, 2020; Turoňová et al., 2020; Figure 1a). Since at least one protomer of *S* homotrimer is in receptor‐accessible conformation, this may be needed to gain entry into the host.

**FIGURE 1:**
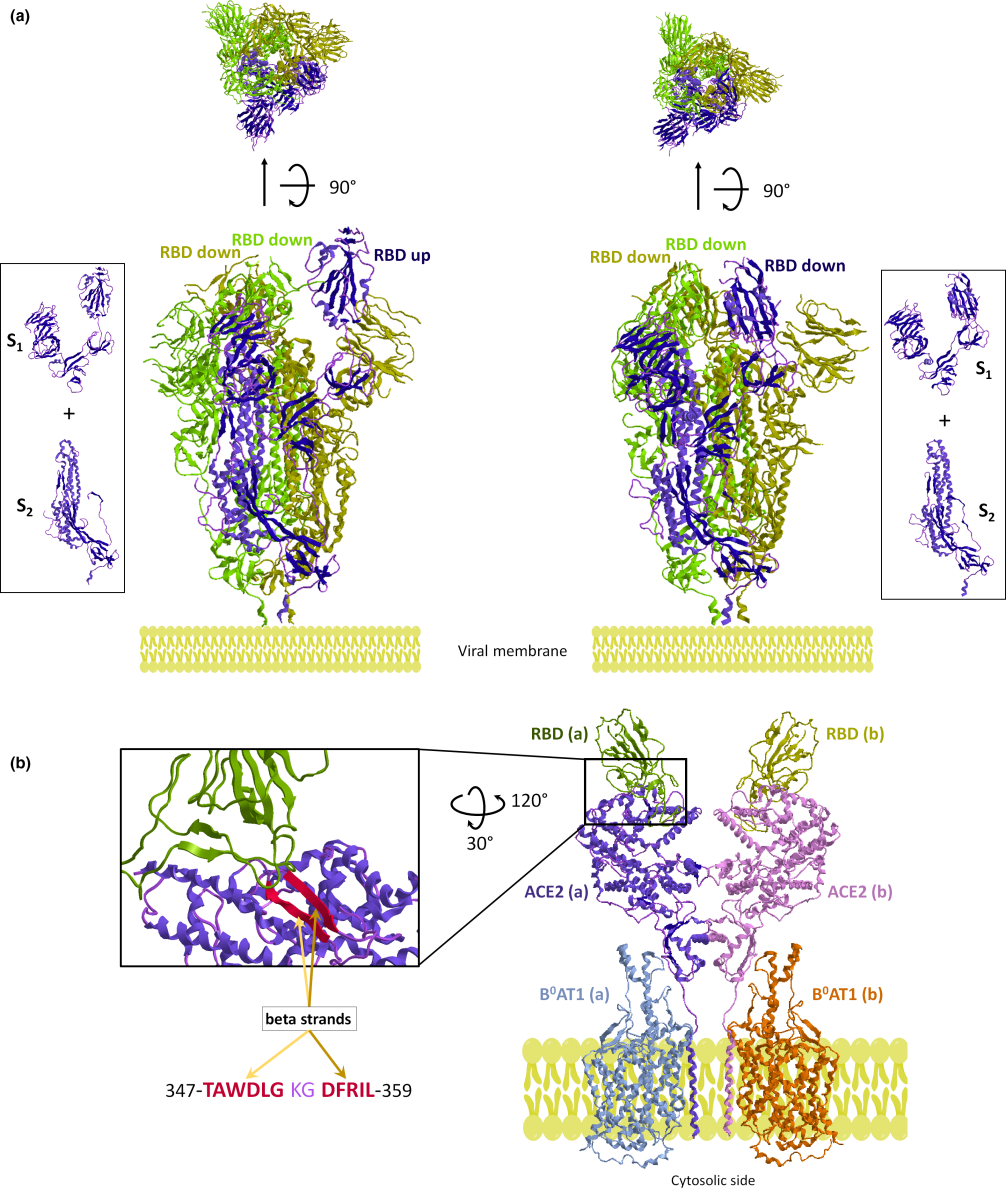
‘Corona’ of SARS‐CoV‐2 and ACE2 engagement. (a) The SARS‐CoV‐2 *S* glycoprotein. The homotrimeric *S* protein in the prefusion conformation with one RBD up conformation (left structure) and an *S* trimer in a non‐accessible conformation with three RBD down (right structure). The side boxes show the subunits (S1 and S2) of an *S* protomer with RBD rotated up (left) and down (right). *S* glycoproteins are drawn as ribbon representations obtained from modified PDB ID codes: 6VSB (left) and 6VXX (right). (b) Structure of the atomic model of the ACE2‐B^0^AT1 complex linked to RBDs of SARS‐CoV‐2. The ribbon representation is obtained from modified PDB ID codes: 6M17. Insert: interactions between SARS‐CoV‐2–RBD up conformation and PD of ACE2. Amino acid sequence of PD beta strands is highlighted in red [Colour figure can be viewed at wileyonlinelibrary.com]

SARS‐CoV‐2 uses the cell entry receptor ACE2 as SARS‐CoV‐1, responsible of SARS2003 (Lan et al., 2020; Zhou et al., 2020). Therefore, *S* glycoproteins establish strong binding interactions with human ACE2 (Mercurio et al., 2021; Turoňová et al., 2020) more efficiently than SARS‐CoV‐1, thereby increasing the ability of SARS‐CoV‐2 to transmit from person to person. ACE2 is an ACE homologue protease expressed in the lungs, heart, kidneys, liver, small intestine and testis. ACE2 has broad substrate specificity acting on the metabolism of the various angiotensin peptides obtained from the renin–angiotensin–aldosterone system. Therefore, ACE2 is implicated in a variety of (un)known pathophysiological processes (Hamming et al., 2007). The monomer of homodimeric ACE2 is a type 1 transmembrane protein with a single transmembrane α‐helix in the C‐terminal region similar to the collectrin domain. The N‐terminal extracellular side consists of the protease domain (PD) with two lobes close to each other after substrate binding, though are in fully open conformation without the substrate (Towler et al., 2004). The syncytia formation between *S* glycoprotein and ACE2 does not occlude the peptidase active site, suggesting that the active site of PD does not interact with RBD of SARS‐CoV‐2 (Li et al., 2005). Interestingly, ACE2 is moonlighting protein (a protein encoded by a single gene that can perform more than one function) which is assembled with B^0^AT1, a member of neutral amino acid transport family, and forms a dimer of ACE2‐B^0^AT1 heterodimers. In other words, two ACE2 proteins in the dimeric form are connected to two monomers of B^0^AT1 (Camargo et al., 2009; Figure 1b). The ACE2 homodimer is structurally possible without B^0^AT1, but the architecture of the complex is not stabilized. However, the B^0^AT1 role is unknown for SARS‐CoV‐2 infection. ‘RBD up’ conformation of SARS‐CoV‐2 directly binds to the PD of the ACE2 monomer, and two trimeric *S* glycoproteins can simultaneously bind to the full‐length human ACE2‐B^0^AT1 complex (Yan et al., 2020). Because ACE2 is a dimer and *S* is a trimer, it might be even speculated that a complete fusion mechanism might involve three ACE2 dimers and two spike trimers, simultaneously (Mercurio et al., 2021).

SARS‐CoV‐2 is unable to use ACE2 proteins from mice as a cellular entry receptor because they are refractory to virus (Zhou et al., 2020). Conversely, overexpression of human ACE2 enhanced disease severity in the mouse model of SARS‐CoV‐2 infection. Thus, the viral entry into target host cells is a critical step. However, the injury was reported to be attenuated by blocking the renin–angiotensin pathway and ACE2 expression (Imai et al., 2005). ACE2 is the molecular key of SARS‐CoV‐2 to facilitate the infection of cells (Yan et al., 2020). Therefore, PD‐ACE2 ‘receptor’ is a potential and scientifically validated therapeutic target to fight the current COVID‐19 pandemic (Zhang et al., 2020). The development of an RBD‐based vaccine is related to the biological interaction between PD‐ACE2 and RBD‐*S* (Lan et al., 2020; Mercurio et al., 2021; Walls et al., 2020; Yan et al., 2020). *S* glycoprotein of SARS‐CoV‐1 and SARS‐CoV‐2 has a high degree of structural homology but different cross‐reactivity of the RBD‐directed monoclonal antibodies (Ahmed et al., 2020). The epitopes of antibodies represent a relatively small percentage of the surface area of interaction into SARS‐CoV‐2 RBD. Therefore, mAB cross‐reactivity could be directed to target a specific RBD region (Lan et al., 2020) or, by considering the two RBD states of *S* in the prefusion conformation, might target a site of an RBD that protrudes toward the central cavity of the *S* protein trimer (Mercurio et al., 2021). Sequence alignments of human and mouse ACE2 have a high level of similarity. Nevertheless, the beta strands, which are highlighted with multiple sequence alignment on human ACE2, are not found in the mouse and rat (Figure 2). In particular, the amino acid sequence *T^347^A^348^W^349^D^350^L^351^G^352^* and *D^355^F^356^R^357^I^358^L^359^*, which form an antiparallel β sheet, is localized on the binding interface between PD‐ACE2 and RBD‐*S* (Figure 1b). Accordingly, the species specificity of SARS‐CoV‐2 mainly relies on the *S*‐receptor interaction with specific structural conformation and/or differences of PD‐ACE2. In particular, the molecular mechanism of host receptor adaptation could be exploited in the development of therapeutic targets (Lu et al., 2020).

**FIGURE 2:**
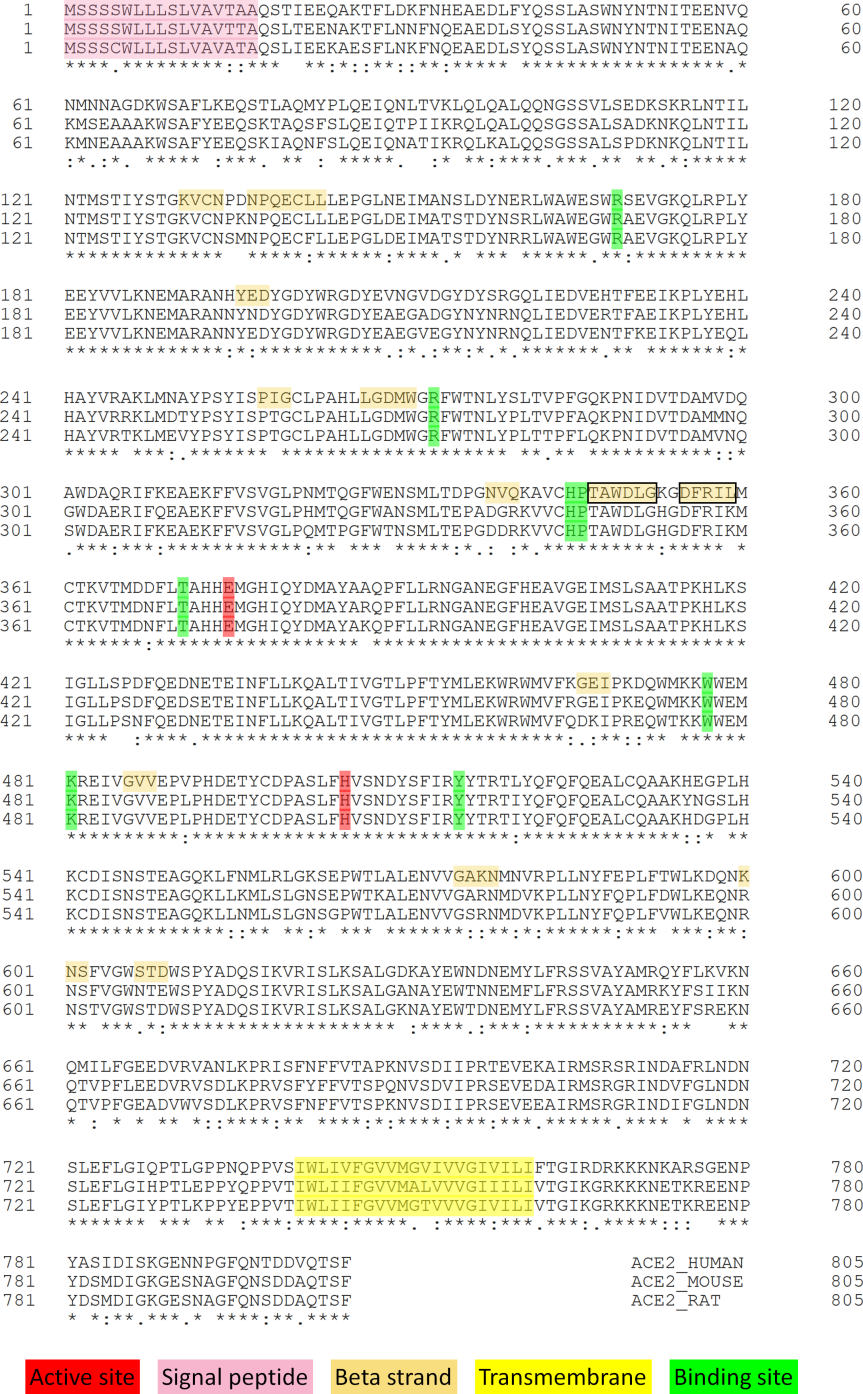
Multiple sequence alignment for ACE2. Entry names of protein sequences: ACE2_HUMAN (*Homo sapiens* accession no. Q9BYF1); ACE2_MOUSE (*Mus musculus* accession no. Q8R0I0); ACE2_RAT (*Rattus norvegicus* accession no. Q5EGZ1). Active site, signal peptide, beta strand, transmembrane and binding site amino acids are highlighted by the colours shown in the respective boxes. The beta strand sequences in the black rectangles located at the level of the RBD‐*S*/PD‐ACE2 interaction region [Colour figure can be viewed at wileyonlinelibrary.com]

The global pandemic situation is a state of emergency due to the presence of the new SARS‐CoV‐2. Therefore, apart from the development of effective vaccines, which represent an alternative approach to fight this pandemic, the design of drugs to counteract all the emerging COVID‐19 virus variants represents a great challenge for the research community.

## CONFLICT OF INTEREST

The author declares no conflict of interest.

## Data Availability

Data sharing not applicable to this article as no datasets were generated or analysed during the current study.
